# Supplementary material for: An integrative framework to prioritize genes in more than 500 loci associated with body mass index
Source: Am J Hum Genet. 2024 May 15;111(6):1035–46. doi: 10.1016/j.ajhg.2024.04.016 (PMC11179420; doi:10.1016/j.ajhg.2024.04.016)
Supplement: Document S1. Figure S1 [file mmc1.pdf]

The American Journal of Human Genetics, Volume *111*

## Supplemental information

# An integrative framework to prioritize genes in more than 500 loci associated with body mass index

**Daiane Hemerich, Victor Svenstrup, Virginia Diez Obrero, Michael Preuss, Arden Moscati, Joel N. Hirschhorn, and Ruth J.F. Loos**

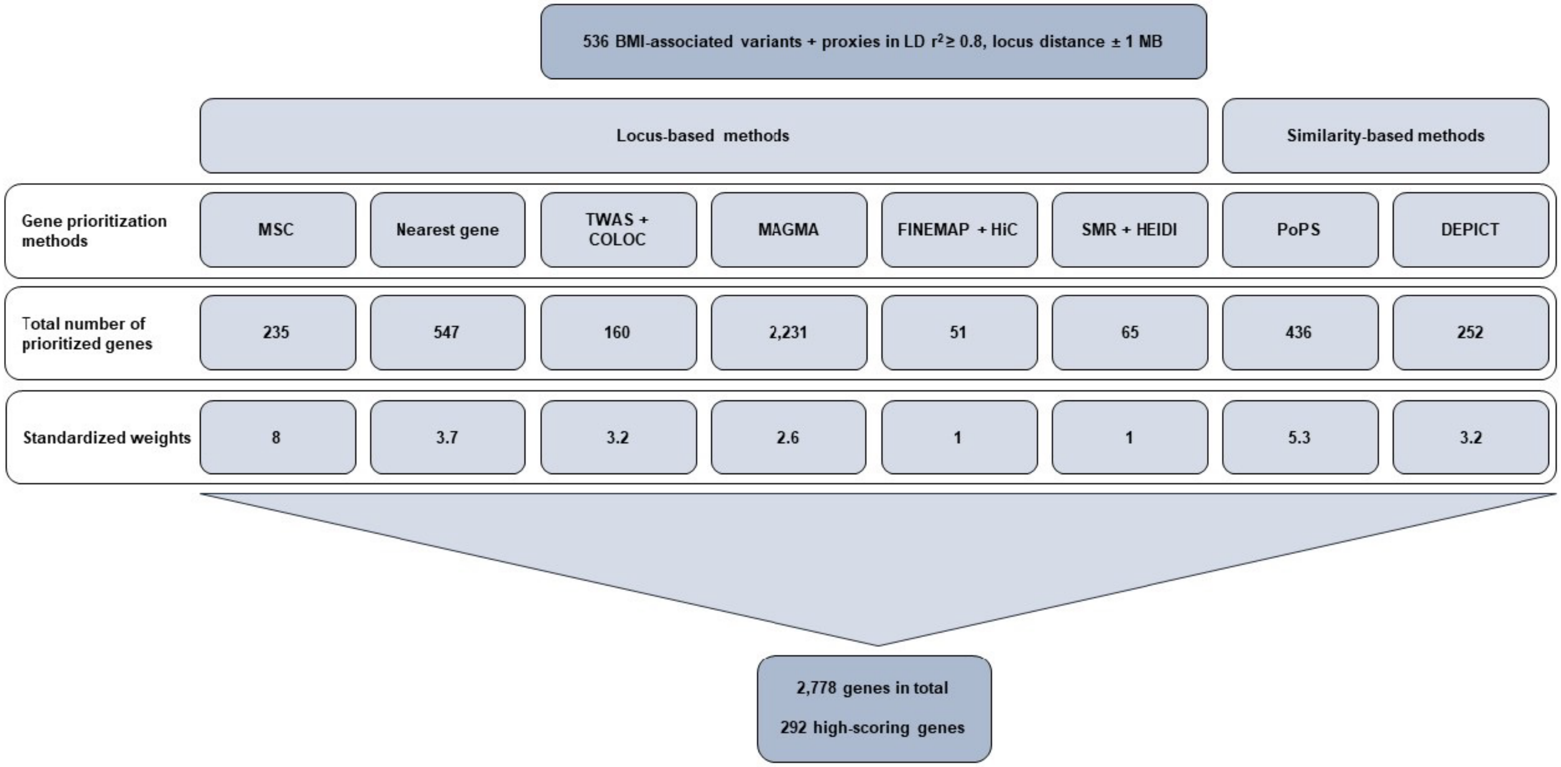


**Figure S1. Flowchart of pipeline, number of genes prioritized and contribution (weights) of each method to the overall scoring of prioritized genes.**
