# Supplementary material for: An integrative framework to prioritize genes in more than 500 loci associated with body mass index
Source: Am J Hum Genet. 2024 May 15;111(6):1035–46. doi: 10.1016/j.ajhg.2024.04.016 (PMC11179420; doi:10.1016/j.ajhg.2024.04.016)
Supplement: Document S2. Article plus supplemental information [file mmc3.pdf]

An integrative framework to prioritize genes in more than 500 loci associated with body mass index

Graphical abstract

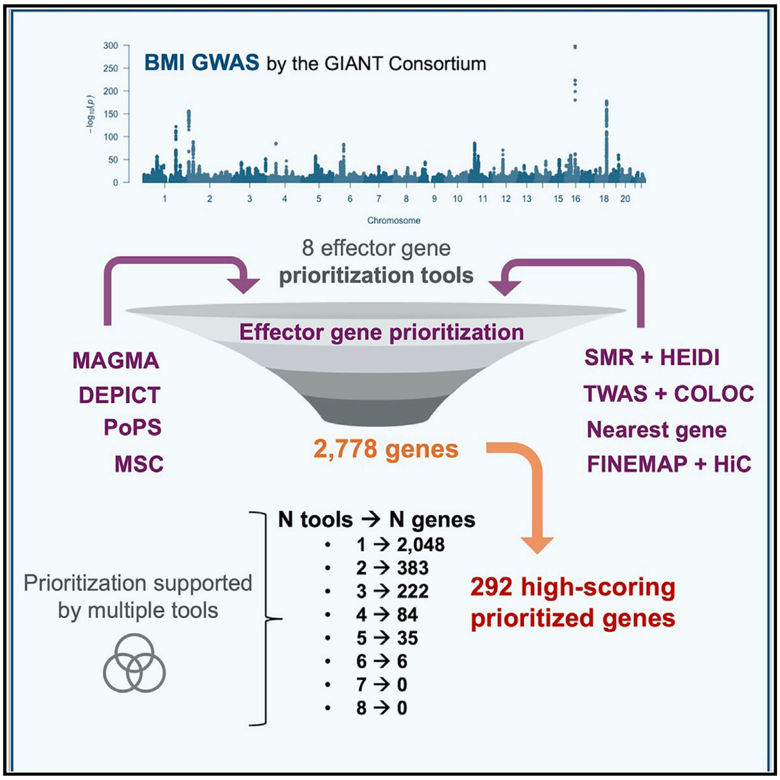

Authors

Daiane Hemerich, Victor Svenstrup, Virginia Diez Obrero, Michael Preuss, Arden Moscati, Joel N. Hirschhorn, Ruth J.F. Loos

Correspondence

ruth.loos@sund.ku.dk

Hemerich et al. apply multiple computational methods to prioritize the likely causal gene(s) within more than 500 previously reported GWAS-identified BMI-associated loci. They identified 292 high-scoring genes, most of which have not previously been implicated in obesity. Characterization of these likely causal genes can provide insights into obesity biology.

Hemerich et al., 2024, The American Journal of Human Genetics *111*, 1035–1046
June 6, 2024 © 2024 The Author(s).
https://doi.org/10.1016/j.ajhg.2024.04.016

CellPress

An integrative framework to prioritize genes in more than 500 loci associated with body mass index

Daiane Hemerich,[1,2] Victor Svenstrup,[3,4] Virginia Diez Obrero,[3,4] Michael Preuss,[1] Arden Moscati,[1,5] Joel N. Hirschhorn,[6,7,8] and Ruth J.F. Loos[1,3,4,*]

Summary

Obesity is a major risk factor for a myriad of diseases, affecting >600 million people worldwide. Genome-wide association studies (GWASs) have identified hundreds of genetic variants that influence body mass index (BMI), a commonly used metric to assess obesity risk. Most variants are non-coding and likely act through regulating genes nearby. Here, we apply multiple computational methods to prioritize the likely causal gene(s) within each of the 536 previously reported GWAS-identified BMI-associated loci. We performed summary-data-based Mendelian randomization (SMR), FINEMAP, DEPICT, MAGMA, transcriptome-wide association studies (TWASs), mutation significance cutoff (MSC), polygenic priority score (PoPS), and the nearest gene strategy. Results of each method were weighted based on their success in identifying genes known to be implicated in obesity, ranking all prioritized genes according to a confidence score (minimum: 0; max: 28). We identified 292 high-scoring genes (≥11) in 264 loci, including genes known to play a role in body weight regulation (e.g., *DGKI*, *ANKRD26*, *MC4R*, *LEPR*, *BDNF*, *GIPR*, *AKT3*, *KAT8*, *MTOR*) and genes related to comorbidities (e.g., *FGFR1*, *ISL1*, *TFAP2B*, *PARK2*, *TCF7L2*, *GSK3B*). For most of the high-scoring genes, however, we found limited or no evidence for a role in obesity, including the top-scoring gene *BPTF*. Many of the top-scoring genes seem to act through a neuronal regulation of body weight, whereas others affect peripheral pathways, including circadian rhythm, insulin secretion, and glucose and carbohydrate homeostasis. The characterization of these likely causal genes can increase our understanding of the underlying biology and offer avenues to develop therapeutics for weight loss.

Introduction

Obesity is a major risk factor for chronic diseases, such as type 2 diabetes, cardiovascular disease, and some cancers.[1] The prevalence of obesity has increased steadily over the past four decades, and most recent reports estimate that nearly 125 million children and adolescents (7%) and more than 670 million adults (13%) worldwide have obesity.[2] Besides contributions of the obesogenic environment, twin and family studies have provided evidence for a genetic component to obesity, with heritability estimates ranging between 40% and 70%.[3,4]

Over the past 15 years, large-scale genome-wide association studies (GWASs) have identified hundreds of genetic loci associated with body mass index (BMI),[5] a commonly used metric to define obesity (BMI $\geq$30 kg/m^2). Pathway, tissue, and functional enrichment analyses, based on the genes located in the GWAS-identified loci, have pointed to the central nervous system (CNS) as a key player in body weight regulation,[6–8] likely influencing hedonic aspects of food intake, such as hunger, satiety, and reward. However, translating GWAS-identified loci into meaningful biology remains a major challenge, as the most significant variant in a locus is often not causal and almost always located in a non-coding region of the genome, likely exerting their effect by acting on genomic elements that regulate the expression of target genes.[9] These so-called effector genes, in turn, are often not in the immediate vicinity of the GWAS locus,[10] and may be regulated through distant interactions with enhancers and looping chromatin.[11,12] This regulatory machinery is highly tissue specific, and for a given locus, effector genes may differ across tissues.[13] Thus, prioritizing effector genes within obesity-associated GWAS loci is a challenging but crucial step to inform functional follow-up experiments that may help us understand the mechanisms that underlie body weight regulation.

Many gene prioritization methods have been developed; they can be divided into locus-based methods and similarity-based methods.[13–18] Combining results across multiple gene prioritization methods has been shown to increase confidence in prioritized genes.[18]

Here, we aim to prioritize genes within each of 536 BMI-associated loci identified in the latest published GWAS meta-analysis by the GIANT Consortium.[19] To this end, we use eight gene prioritization methods, including

[1]The Charles Bronfman Institute for Personalized Medicine, Icahn School of Medicine at Mount Sinai, New York, NY, USA; [2]Bristol Myers Squibb, Summit, NJ, USA; [3]Novo Nordisk Foundation Center for Basic Metabolic Research, University of Copenhagen, Copenhagen, Denmark; [4]Novo Nordisk Foundation Center for Genomic Mechanisms of Disease, Broad Institute of MIT and Harvard, Cambridge, MA, USA; [5]Regeneron Genetics Center, Tarrytown, NY, USA; [6]Department of Genetics, Harvard Medical School, Boston, MA 02115, USA; [7]Program in Medical and Population Genetics, Broad Institute of Harvard and MIT, Cambridge, MA 02142, USA; [8]Division of Endocrinology and Center for Basic and Translational Obesity Research, Boston Children's Hospital, Boston, MA 02115, USA
*Correspondence: ruth.loos@sund.ku.dk
https://doi.org/10.1016/j.ajhg.2024.04.016.
© 2024 The Author(s). This is an open access article under the CC BY license (http://creativecommons.org/licenses/by/4.0/).

locus-based and similarity-based methods. We score and rank the prioritized genes according to the ability of each method to identify established obesity genes.[20,21] As such, we generate a catalog of candidate causal genes (i.e., a gene that likely harbors a causal variant), prioritized in each GWAS-identified obesity locus. A weighted score was calculated for each candidate gene, based on the type and number of prioritization methods that prioritized it. This catalog may expedite the selection of candidate genes for functional characterization in experimental follow up studies, critical to bridge the translational gap—from variant to function—which has been lacking in most GWASs.

Material and methods

GWAS-identified BMI-associated loci

We obtained publicly available GWAS summary statistics from Yengo et al.[19] from https://portals.broadinstitute.org/collaboration/giant/index.php/GIANT_consortium. Their combined GWAS meta-analysis includes $N = \sim 700{,}000$ individuals. Loci were defined as one or multiple jointly associated SNPs located within a 2 Mb window (± 1 Mb of the lead SNP).

Gene prioritization methods

SMR analysis

Briefly, the SMR and HEIDI approach integrates summary-level data from GWASs and eQTL studies to test whether a transcript and phenotype are associated because of a shared causal variant (i.e., pleiotropy). The advantage of SMR when compared with similar integrative approaches[22–24] is the ability to distinguish a pleiotropic model (i.e., gene expression and phenotype are associated owing to a single shared genetic variant) from a linkage model (i.e., there are two or more distant genetic variants in LD affecting gene expression and phenotype independently).[25] We considered as candidate genes those passing a Bonferroni corrected p-SMR and a p-HEIDI < 0.05, as in similar studies.[25]

LD data required for the HEIDI test were estimated from genotyped data from the UK Biobank (UKB) study,[26] including 10,000 randomly selected white British participants (Project 1251; https://biobank.ndph.ox.ac.uk/ukb/label.cgi?id=263). Appropriate informed consent was obtained from the participants.

To map the resulting genes to their respective BMI-associated loci, we identified the lead BMI SNP in high LD ($r^2 > 0.8$) with the top SNP that passed SMR and HEIDI tests.

Blood eQTL summary statistics were obtained from eQTLGen Consortium,[27] generated on peripheral blood from 31,684 individuals. SMR-formatted data were downloaded from https://molgenis26.gcc.rug.nl/downloads/eqtlgen/cis-eqtl/SMR_formatted/cis-eQTL-SMR_20191212.tar.gz. We used different brain eQTL summary datasets, including GTEx-brain ($n = 72$),[28] Common Mind Consortium (CMC) ($n = 467$),[29] ROSMAP ($n = 494$),[30] and Brain-eMeta ($n = 1{,}194$).[28] Data from GTEx-brain was generated via MeCS method by Qi et al.[28] to account for sample overlap, given brain data available on GTEx comes from 10 brain regions.[28] CMC and ROSMAP eQTL data for SMR analyses were obtained from Qi et al.[28] Brain-eMeta was generated in the same study by the MeCS method and is a meta-analysis of GTEx brain, CMC, and ROSMAP.[28] Both CMC and ROSMAP eQTLs were from a larger set of dorsolateral prefrontal cortex tissue samples. We downloaded SMR-formatted GTEx, CMC, ROSMAP, and Brain-eMeta from https://yanglab.westlake.edu.cn/software/smr/#DataResource.

DEPICT analysis

DEPICT is an integrative tool that prioritizes the most likely causal genes based on predicted gene functions and identifies enriched pathways, tissues/cell types in which the presumed causal genes are expressed.[17] We used BMI summary statistics from Yengo et al.[19] (https://portals.broadinstitute.org/collaboration/giant/index.php/GIANT_consortium) as input on DEPICT with default parameters. DEPICT is built upon 14,461 predefined gene sets from diverse databases and data types and applies a stepwise approach that consists of the scoring, bias adjustment, and FDR estimation steps. Briefly, it first scores the similarity of a given gene with genes of the 14,461 gene sets by applying a correlation approach. Next, it controls for biases, such as gene length, by normalizing the gene score. Finally, FDRs are estimated by repeating the previous two steps (scoring and bias adjustment) 20 times, based on top SNPs from the precomputed null GWAS.

FINEMAP analysis and chromatin conformation mapping

We performed a GWAS on BMI using data of 452,956 European UK Biobank participants (Project 1251; https://biobank.ndph.ox.ac.uk/ukb/label.cgi?id=263), using the same criteria and methods as described in Yengo et al.[19] and used BMI summary statistics as input on FINEMAP[31] with default parameters and selecting a maximum of 30 causal variants per locus. The output variants identified as likely causal were mapped to genes using tissue-specific HiC chromatin conformation capture data.[32] We integrated all HiC data in brain (dorsolateral prefrontal cortex, hippocampus, neural progenitor cell, adult and fetal cortex, temporal cortex, and cerebellum) available on FUMA v.1.3.5,[33] using the aforementioned tool (https://fuma.ctglab.nl/). The data available in FUMA are available at GEO: GSE87112.[34] We also used FUMA to integrate chromosomal conformation capture data on neurons, microglia, and oligodendrocytes from Nott et al.,[35] which is available at dbGaP (accession number phs001373.v2.p2).

Potentially damaging variants

We investigated variants whose amino acid change can lead to a potentially damaging effect. To retrieve variants in LD with the lead associated SNPs, we used FUMA v.1.3.6a[33] with parameters $r^2 > 0.8$ and MAF > 0, using UKB release 2b 10k European as the reference panel (https://fuma.ctglab.nl/snp2gene). We used the variants output on FUMA as input on the Mutation Significance Cutoff (MSC) web server (https://itanlab.org/resources/software/),[36] selecting CADD 1.3 (https://cadd.gs.washington.edu/download)[37] and database HGMD (https://www.hgmd.cf.ac.uk/ac/index.php).[38] Genes whose prediction by MSC with 95% confidence interval of having a high damaging impact were prioritized. We retrieved minor allele frequencies (MAFs) from Ensembl Biomart using Ensembl Variation 104 Human Short Variants GRCh37.13 database (https://grch37.ensembl.org/biomart/martview/8562f843c754417502c69dc46005d6dc), selecting the "Global minor allele frequency (all individuals)" option.

Gene-based analysis with MAGMA

We run gene-based analysis with MAGMA v.1.8 (https://cncr.nl/research/magma/),[39] using BMI summary statistics from Yengo et al.[19] and a reference panel of 10K randomly selected white British individuals from the UK Biobank (Project 1251; https://biobank.ndph.ox.ac.uk/ukb/label.cgi?id=263). We performed MAGMA on a gene window of 100 KB and applied the Bonferroni correction as multiple-testing correction method to obtain the most significant results.

Transcriptome-wide association study

We used FUSION to identify genes whose *cis*-regulated expression is associated with BMI through a transcriptome-wide association study (TWAS) (http://gusevlab.org/projects/fusion/).[24] For a given gene, a TWAS uses eQTL data to train a gene expression prediction model that will "impute" the expression across a large cohort of genotyped individuals, followed by a test of association with a given trait of disease risk. The TWAS may additionally increase power versus single SNP association testing, either by reducing the multiple testing burden or aggregating multiple expression-altering variants into a single test.[40] In our analysis, we included pre-computed gene expression weights generated in tissues specific to BMI (brain data from the CMC study[29] and GTEx v.7 data on brain amygdala, anterior cingulate cortex, caudate, cerebellar hemisphere, cerebellum, cortex, frontal cortex, hippocampus, hypothalamus, nucleus accumbens, putamen, spinal cord and substantia nigra), excluding the MHC region (we downloaded FUSION-ready preprocessed data from http://gusevlab.org/projects/fusion/). We used the COLOC module available on FUSION to colocalize significant TWAS associations with eQTL data and retrieved only significant results at PP4 > 0.8. To map the resulting genes to their respective BMI-associated loci, we identified the lead BMI SNP in high LD ($r^2 > 0.8$) with the GWAS SNP that passed TWAS and COLOC tests.

Polygenic Priority Score

Polygenic priority score (PoPS) is built upon data from an extensive set of bulk and single-cell expression datasets, curated biological pathways, and predicted protein-protein interactions.[18] It assigns a priority score to every protein-coding gene according to enrichments with these datasets. We used Polygenic Priority Score v.0.1 (https://github.com/FinucaneLab/pops) with a reference panel of 10,000 randomly selected subjects from the UKB (Project 1251; https://biobank.ndph.ox.ac.uk/ukb/label.cgi?id=263). We retrieved the gene with the highest PoPS score in each BMI-associated loci.[18]

Scoring and ranking genes

Nine gold standard obesity genes from Hendricks et al.[20] and Marenne et al.[21] were used to score the eight prioritization approaches. These obesity genes are *LEPR, POMC, PCSK1, LEP, SH2B1, MC4R, PHIP, DGKI,* and *ZMYM4*. Of these, six are located in BMI-associated loci (1 MB each side of a lead BMI-associated variant): *LEPR, POMC, PCSK1, DGKI, SH2B1,* and *MC4R*. We counted how many of these six established obesity genes each method was able to identify in the BMI-associated loci (Figure 2). We calculated the proportion of genes identified by each method by dividing the number of gold standard genes found by the total number of genes found by the method within BMI-associated loci. We next normalized this proportion to a scale of 1–8 (given there are eight gene prioritization method). The normalized proportions are the final score of each method (Table 1). Genes were ranked by the sum of the scores relative to the method by which they were identified. With this system, we prioritized the top 292 high-scoring genes (top 10%, score >11) as our final list of genes likely implicated in BMI.

The visualization of number of overlapping genes in Figure 1 was generated using package UpSetR.[41]

Genes targeted by enhancers

We used predicted enhancers and their target genes in 131 cell types from Nasser et al.[42] and 32 cell types from Boix et al.[43] (data available on https://personal.broadinstitute.org/cboix/epimap/links/pergroup/). We examined the overlap between the lead BMI-associated variants and their proxies ($r^2 > 0.8$) of the 292 high-scoring candidate genes with enhancers, to assess the presence of prioritized genes that are potentially regulated by BMI variants overlapping these tissue-specific regulatory elements.

Pathway enrichment analyses

We performed pathway enrichment analyses based on the 292 high-scoring candidate genes using the Gene Ontology (GO) database, which contains structured biomolecular annotations that indicate biological processes, molecular functions, or cellular components. This analysis assessed the over/under-representation of the set of 292 prioritized genes in the curated gene-sets at the GO database. This analysis was performed with the FUMA (v.1.3.5) (https://fuma.ctglab.nl/).[33]

Software and data used

All software and data used in this paper are publicly available. Links to the software used is in the "Access" column of Table 1. Usage of data is in compliance with the data use agreements of each respective source.

Results

Eight gene prioritization methods implicate 2,778 genes across 536 BMI-associated loci

Using six locus-based methods (nearest gene, MAGMA, FINEMAP+HiC, MSC, TWAS+COLOC, SMR+HEIDI) and two similarity-based methods (DEPICT, PoPS), we prioritized 2,778 genes across the 536 BMI-associated loci (material and methods, Table 1; Figures 1 and 2; Table S1).

MAGMA, a gene analysis method to detect multi-marker effects,[39] prioritized the most candidate genes (N_{genes} = 2,231) (material and methods, Tables 1, S1, and S2).

The mutation significance cutoff (MSC) method prioritizes genes based on the damaging impact of lead variants or their proxies located in those genes[36] (material and methods). With this approach, we prioritized 235 genes in which lead variants or proxies had a predicted high damaging impact (Tables S1 and S3), of which 20 variants in 10 genes (*DNALI1*, *GNL2*, *GRID1*, *GPR61*, *ISL1*, *MC4R*, *SLC39A8*, *SNIP1*, *TNRC6C*, and *UBAP2*) are of low frequency (minor allele frequency [MAF] < 5%), of which one (in *GPR61*) was rare (MAF < 1%) (material and methods, Tables 1, S1, and S3).

The nearest gene method consists of retrieving the protein-coding gene nearest to the lead variant. It is a common and simple strategy for gene prioritization and is considered reasonably effective.[18] We identified 547 genes that are near or overlapping the 536 lead BMI-associated variants (material and methods, Tables 1 and S1), with twelve variants overlapping with more than one gene. Of the 536 variants, 186 are intergenic, whereas the others are either intronic ($N = 320$), exonic ($N = 16$), or fall within an untranslated region, UTR3 ($N = 11$) or UTR5 ($N = 3$), of one, and sometimes more, genes. The average distance to the nearest gene is 98,200 bp, with the furthest gene

Table 1. Methods used for the gene prioritization, and number of genes they prioritize

Method	Description	Access	Reference	# of genes prioritized in the 536 BMI-associated loci	# of established obesity genes identified (max 6)[a]	% of established genes identified relative to total genes prioritized	Standardized weights
**Locus-based methods**							
Mutation significance cutoff (MSC)	provides gene-level and gene-specific phenotypic impact cutoff values, as opposed to a single significance cutoff value across all genes	https://lab.rockefeller.edu/casanova/MSC	Itan et al.[36]	235	3	1.30%	8
Nearest gene	gene nearest to lead variant	–	–	547	3	0.50%	3.7
Transcriptome-wide association study (TWAS) + COLOC	TWAS leverages expression imputation (pre-computed gene expression weights generated from individuals for whom both gene expression and genetic variation have been measured) to test for significant genetic correlation between *cis* expression and GWAS; the imputed expression can be viewed as a linear model of genotypes with weights based on the correlation between SNPs and gene expression in the training data while accounting for LD among SNPs; COLOC further estimates the posterior probability of colocalization, where colocalization is defined as one (or more) shared causal variants between the expression and GWAS	http://gusevlab.org/projects/fusion/	Gusev et al.[24] & Giambartolomei et al.[22]	160	1	0.60%	3.2
Multi-marker analysis of GenoMic annotation (MAGMA)	MAGMA first computes a gene-based *p* value based on the mean association of variants in the gene, accounting for LD between variants; then, competitive gene-set and/or continuous covariate *p* values are calculated, based on the association of the gene-based *p* values with the category of interest	https://ctg.cncr.nl/software/magma	de Leeuw et al.[39]	2,231	6	0.30%	2.6
FINEMAP + HiC	FINEMAP uses a Bayesian approach to determine which are the most likely causal variants in a locus; candidate causal variants identified by FINEMAP are mapped to genes using chromatin conformation capture HiC data, which represents loops of DNA where regions of the genome interact	http://www.christianbenner.com	Benner et al.[31]	51	0	0%	1

(*Continued on next page*)

Table 1. *Continued*

Method	Description	Access	Reference	# of genes prioritized in the 536 BMI-associated loci	# of established obesity genes identified (max 6)[a]	% of established genes identified relative to total genes prioritized	Standardized weights
Summary-data-based Mendelian randomization (SMR) + heterogeneity in dependent instruments (HEIDI)	integrates summary-level data from GWASs with data from eQTL studies to identify genes whose expression levels are associated with a complex trait because of pleiotropy; the methodology can be interpreted as an analysis to test if the effect size of an SNP on the phenotype is mediated by gene expression; the HEIDI method then uses multiple SNPs in a *cis*-eQTL region to distinguish pleiotropy from linkage	https://cnsgenomics.com/software/smr/	Zhu et al.[25]	65	0	0%	1
**Similarity-based methods**							
Polygenic priority score (PoPS)	uses gene-level associations computed from GWAS summary statistics to learn joint polygenic enrichments of gene features derived from gene expression, biological pathways, and protein-protein interactions (PPI), assigning a priority score to every protein-coding gene	https://github.com/FinucaneLab/pops	Weeks et al.[18]	486	4	0.80%	5.3
Data-driven expression prioritized integration for complex traits (DEPICT)	employs annotated gene sets (including manually curated pathways, molecular pathways from protein-protein interaction screens, and phenotypic gene sets from mouse gene knock-out studies). By calculating, for each gene, the likelihood of membership in each gene set (based on similarities across expression data), 14,461 'reconstituted' gene sets were generated. Using these precomputed gene functions and a set of trait-associated loci, DEPICT assesses whether any of the 14,461 reconstituted gene sets are significantly enriched for genes in the associated loci, and prioritizes genes that share predicted functions with genes from the other associated loci more often than expected by chance.	https://github.com/perslab/depict	Pers et al.[17]	252	1	0.40%	3.2

[a]"Established genes" refers to the nine gold standard obesity genes from Hendricks et al.[20] and Marenne et al.[21] (*LEPR, POMC, PCSK1, LEP, SH2B1, MC4R, PHIP, DGKI,* and *ZMYM4*).

(*ADGRL3*) being 1.8 Mb away from the lead variant (rs925421).

In the combined transcriptome-wide association study (TWAS) and COLOC method, TWAS integrates predicted gene expression levels with GWAS summary statistics to identify genes whose *cis*-regulated expression is associated with a complex trait,[24] whereas COLOC tests the colocalization of the association signals.[22] As such, we identified 160 genes across the 536 loci for which the BMI-associated lead SNP or a proxy variant is associated with its gene expression across different tissues (material and methods, Tables 1, S1, and S4). Since tissues related to the central nervous system have been shown to be enriched among GWAS-identified BMI loci, we focused on eQTL datasets from brain tissue.

In the combined summary-data-based Mendelian randomization (SMR) and HEIDI method, we test whether the association between the lead variant (or its proxy) and BMI is mediated through an eQTL of a gene nearby.[25] As we did for a TWAS, we focused on eQTL datasets from brain tissue, but we also considered data from blood, for which datasets generated from bigger sample sizes were available (material and methods). This approach prioritized 27 genes in blood and 43 genes in brain tissue. Five genes were prioritized in both tissues (Tables 1, S1, and S5), resulting in a total of 65 prioritized genes.

Using FINEMAP,[31] a Bayesian approach to pinpoint the likely causal variant(s) in a locus, we prioritized 81 candidate causal variants (material and methods, Tables 1, S1, and S6). We were able to map 26 variants to 51 genes using brain tissue-specific HiC chromosomal conformation data (material and methods). This approach showed the least overlap with other approaches, with only 20 (39%) of the 51 genes also being identified by other approaches (Figure S1).

Finally, we used DEPICT and PoPS, two similarity-based algorithms for gene prioritization. DEPICT (data-driven expression-prioritized integration for complex traits),[17] which aims to systematically prioritize the most likely causal genes in a locus based on predicted gene functions, prioritized 252 candidate causal genes (material and methods, Tables 1, S1, and S7). PoPS (polygenic priority score),[18] which leverages both polygenic and locus-specific genetic signals by combining results across multiple gene prioritization methods, identified 486 genes (material and methods, Tables 1, S1, and S8). PoPS integrates more layers of information on gene expression, generated using next-generation sequencing techniques, compared to DEPICT, and where PoPS uses genome-wide summary statistics, DEPICT uses only summary statistics in the genome-wide significant loci.

The eight prioritization methods combined prioritized 2,778 unique genes across the 536 BMI-associated loci (Figures 1 and S1). While no genes were prioritized by seven or more methods, six genes were prioritized by six methods (*ANKRD26*, *BPTF*, *GGNBP2*, *KAT8*, *YWHAZ*, and *ZNF131*) and 35 genes were prioritized by five methods (Figure 1; Table S9).

Ranking of prioritized genes and catalog of obesity genes

We next built a prioritization score that weighs each of the eight methods based on whether or not they prioritized one or more of the six established obesity genes located in any of the 536 BMI-associated loci (i.e., *LEPR, POMC, PCSK1, DGKI, SH2B1,* and *MC4R)*. MAGMA identified all six, while PoPS identified four, MSC and the nearest gene strategy each identified three, TWAS and DEPICT each identified one, and SMR and FINEMAP did not identify any of the six established obesity genes and was given the lowest priority weight of 1 (Table 1). We calculated the percentage of established genes that were prioritized by a given method, relative to the total number of genes prioritized across all BMI-associated loci (Table 1). We then converted these percentages into a continuous scale of 1–8, consistent with the number of prioritization methods used (Table 1). Next, we assigned a prioritization score to each of the 2,778 genes implicated by the eight prioritization methods by summing the weight of each method by which the gene had been prioritized (Table S9).

The prioritization score across the 2,788 genes ranged from 1 to 24.8, whereas the theoretical max, i.e., when a gene is identified by all 8 methods, is 28. The average score is 5; 292 (10.5%) of the 2,788 prioritized genes scored more than 11, of which 99 (3.6%) scored more than 15 (Table S9). Two genes (*ANKRD26* and *BPTF*) reached the highest prioritization score (24.8). Several of the high-scoring genes (score $\geq$ 11) are known to be implicated in obesity (such as *DGKI* [score: 22.8], *MC4R* [19.6], *BDNF* [19.6], *MTOR* [16.8], *SH2B1* [14.8], *GIPR* [14.3], *AKT3* [11.6], and *LEPR* [11.6]). For other high-scoring genes, there is mounting evidence, but more research is needed to establish their role (such as for *ANKRD26* [24.8], *NPC1* [23.8], *NCOR1* [22.8], *BMAL1* [22.8], *KAT8* [22.7], *GSK3B* [20.2], *ISL1* [19.6], *PRKN* [19.6], *MST1R* [18.5], *PDS5B* [17.5], *FGFR1* [17], and *VPS13C* [15.9]). However, for many other high-scoring genes, a role in body weight regulation remains to be determined.

We also tested whether the BMI-associated variants or their proxies overlap with an enhancer predicted to target the 292 high-scoring genes. In total, BMI-associated variants (and their proxies) in/near 217 of the 292 prioritized genes overlap enhancers (material and methods, Table S10), suggesting potential mechanisms by which these genes are affected in BMI-associated loci. Of the 292 high-scoring genes, 135 link to BMI-associated variants that overlap enhancers in the brain, the main tissue implicated in obesity-associated loci.

Pathway enrichment analyses applied to the 292 high-scoring prioritized genes implicate gene sets and pathways related to the central regulation of body weight (material and methods, Table S11), such as the neurotrophin signaling pathway[44] (including *BDNF, AKT3, RPS6KA5, MAP2K5, SH2B1, MAP3K3, BCL2, GSK3B, FOXO3, RAC1*, and *YWHAZ*), which regulates appetite,[45] and the PI3K/AKT pathway (including *AKT3, FOXO3, GSK3A, GSK3B,*

MTOR, and *YWHAZ*),[46] which is involved in central and peripheral appetite regulation and is implicated in the development of insulin resistance in peripheral tissues.[47] Besides the many pathways that act in the brain, other enriched pathways and gene sets implicate circadian rhythm (*BMAL1*, *NCOR1*, *PPP1CB*, *ZFHX3,* and *HDAC3*), insulin secretion (*HMGB1*, *MTOR*, *PDE1C*, *CADPS*, and *ZBTB20*), adipocyte differentiation (*NCOR1*, *KAT8*, *KDM4C*, *CCDC 171*, *PPP1CB*, *RPS6KA5*, *HMGB1*, *VPS13C*, and *ESRRA*), and glucose and carbohydrate homeostasis (*MAP4K4*, *GSK3B*, *PPP1CB*, *MAP2K5*, *FGFR1*, *MTOR*, *CREB1*, and *GSK3A*) in the control of body weight.

Discussion

Using a broad range of eight gene prioritization methods, we identified 2,778 unique genes across 536 BMI-associated loci prioritized by at least one method. We ranked these candidate genes based on the number of methods that prioritized a given gene, weighted by the ability of each method to identify established obesity genes in GWAS loci. We prioritized 292 high-scoring candidate genes for obesity, enriched in neuron-related pathways, synapses, signaling, and behavior, but also genes implicated in peripheral biology and expressed in metabolically active tissues.

We found several high-scoring genes for which extensive evidence on their role in obesity already exists, such as *MC4R*,[48–51] *BDNF*,[52–54] *GIPR*,[55–57] *DGKI*,[21] *MTOR*,[58,59] *AKT3*,[60] and *LEPR*[61,62] (Table S9). For other genes, a link with body weight regulation was available, but more research to further establish them as obesity genes is needed. For example, *KAT8*,[63] *KDM4C*,[64] *PPP1CB*,[65] and *RPS6KA5*[66] have been linked to adipocyte differentiation (Table S9).

For most other high-ranking genes, however, the current evidence for a link with obesity is weak or non-existent. Nevertheless, some of these genes with weaker evidence were also prioritized in two other studies that aimed to identify candidate genes in BMI-associated loci, providing independent supporting evidence.[67,68] The first study, which focused on 97 BMI-associated genes (a subset of the 536 GWAS loci studied here) of an earlier GWAS meta-analysis, established a functional genomics pipeline that integrates a comprehensive regulatory map in adipose and hypothalamic neurons and a massively parallel assay to connect each of the 97 lead variants to putative candidate genes.[67] Eight of the 19 genes that scored high with the functional genomics pipeline also scored high using our integrated bioinformatic approach; these genes include *NPC1* (score: 23.8), *CCDC171* (19.5), *MAP2K5* (17.5), *SH2B1* (14.8), *NUP88* (14.3), *POC5* (14.3), *TUFM* (11.8), and *ATXN2L* (11.1). The second study, which focused on the same BMI-associated loci as in our study, built a pipeline to map non-coding variants with nearby effector genes by integrating chromatin structure and transcriptomics data at three developmental stages during hypothalamic differentiation.[68] Of the 67 genes implicated for BMI, nine overlapped with high-scoring genes in our study, including *MLLT10* (22.8), *BDNF* (19.6), *SETBP1* (19.6), *FGFR1* (17), *CAST* (14.3), *TUFM* (11.8), *GABRB3* (11.6), *MAD1L1* (11.6), and *THRA* (11.1).[68] Even though for most of these genes, their role in body weight regulation is unknown, the convergence of evidence across the two prioritization approaches strengthens their candidacy in the locus.

Other high-scoring genes for which the connection to obesity and body weight regulation remains to be determined include *BPTF*[69] (score: 24.8), *RSRC1*[70,71] (22.8), and *AUTS2*[72] (19.6), three genes in which mutations or chromosomal rearrangements have been linked to intellectual disability and neurodevelopmental anomalies. Others among the top 10% high-scoring genes with an unknown role in the context of obesity include *ERC2* (23.8), *ZNF131* (23.8), *NLGN1* (22.8), *MLTT10* (22.8), *RERE* (22.8), and *PDCH9* (22.8). Investigating the role of these genes in the pathophysiology of obesity might reveal regulatory pathways in obesity, some of which may provide new anti-obesity therapeutic targets.

Pathway enrichment analyses highlight the central nervous system as a key organ in body weight regulation, which is consistent with previous observations,[6–8] but this might be influenced by the fact that for three of the prioritization methods (TWAS/Coloc, SMR, and FINEMAP), the underlying molecular datasets we used were solely from the brain. Nevertheless, we believe the potential bias is limited by the fact that these three prioritization methods contributed the least to the scoring of prioritized genes. Furthermore, several of the high-scoring genes have been implicated in peripheral pathways and gene sets, related to adipocyte differentiation (*NCOR1* [22.8], *KAT8* [22.8], *KDM4C* [19.6], *CCDC171* [19.5], *PPP1CB* [19.0], *RPS6KA5* [17.5], *HMGB1* [17.5], *VPS13C* [15.9], and *ESRRA* [14.3]), circadian rhythm (*BMAL1* [22.8], *NCOR1* [22.8], *PPP1CB* [19], *ZFHX3* [14.8], and *HDAC3* [11.1]), insulin secretion (*HMGB1* [17.5], *mTOR* [16.8], *PDE1C* [15.8], *CADPS* [14.8], and *ZBTB20* [14.8]), and glucose and carbohydrate homeostasis (*MAP4K4* [22.8], *GSK3B* [20.2], *PPP1CB* [19], *MAP2K5* [17.5], *FGFR1* [17], *MTOR* [16.8], *CREB1* [11.6], and *GSK3A* [11.1]). The ability to capture known pathways involved in the pathophysiology of obesity suggests that our list of ranked prioritized genes are likely enriched for true effector genes and can therefore be used to identify regulatory pathways implicated in the pathophysiology of obesity and its comorbidities.

For 29 loci, at least two prioritized genes were high scoring (Table S9). For example, in the locus of lead BMI-associated SNP rs1549293, *KAT8* (22.7), the nearest gene, *ZNF646* (14.8), and *ZNF668* (11.6) were prioritized. Other examples of loci that with multiple high-scoring genes include the loci represented by rs1075901 with *NCOR1* (22.8) and *TTC19* (13.7), rs1106908 with *GGNBP2* (22.7) and *DHRS11* (15.8), and rs8075273 with *MAP3K3* (21.7)

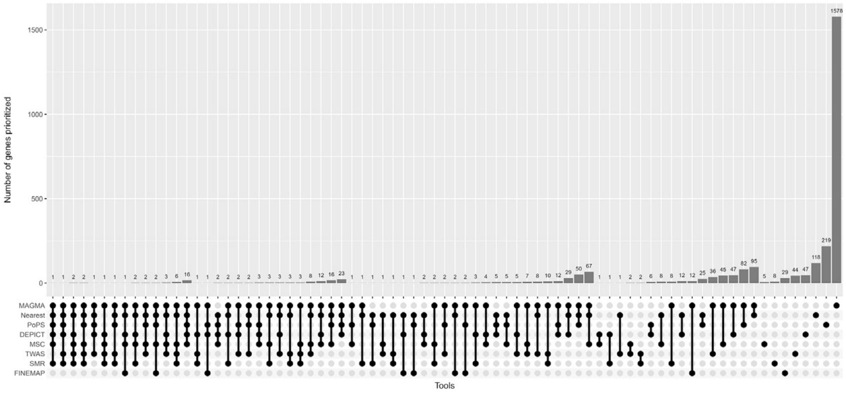

Figure 1. Number of genes prioritized in 536 BMI-associated loci by the eight methods
Right side shows one method only; far left shows six methods at the same time. Y-axis shows number of prioritized genes overlapping between the methods.

and *DDX42* (15.8). These results highlight that in a given locus, more than one gene can be causal, as is the case for the *FTO* locus for which a comprehensive analysis of the genetic and functional architecture showed that multiple variants in the locus overlap with enhancers that target *IRX3* and *IRX5*.[73]

Our gene prioritization pipeline also identified genes encoding known therapeutic targets for obesity and its comorbidities, such as the glucose-dependent insulinotropic polypeptide (GIP) receptor, *GIPR* (14.3), and the fibroblast growth factor 21 (*FGF21*) co-receptor, *FGFR1* (17), and a gene encoding a subunit in the NMDA receptor, *GRIN3A* (14.3). The GIPR, together with the glucagon-like peptide 1 receptor (GLP-1R), is a target of the incretin dual-agonist, tirzepatide. This drug has been shown in clinical trials to provide substantial and sustained weight loss in individuals suffering from obesity.[74,75] In addition, it is FDA approved for the treatment of type 2 diabetes. Pharmaceutical companies are currently evaluating FGFR1 agonists and FGF21 analogous for the treatment of dyslipidemia and non-alcoholic steatohepatitis (NASH), which have been shown to reduce body weight, improve lipid profiles, reduce liver fat content, and reduce liver fibrosis in individuals with NASH, and resolve NASH in clinical trials.[76–78] *GRIN3A* encodes a subunit in the NMDA receptors, which is implicated in appetite and food preference regulation.[79] The NMDA antagonist, memantine, has been shown to reduce body weight in preclinical studies.[80] The ability of our gene prioritization approach to identify genes encoding well-known therapeutic targets for the treatment of obesity and its comorbidities highlights the opportunity to identify anti-obesity therapeutics targets using this integrative framework of gene prioritization methods.

Our study demonstrates the value of the integration of a range of methods for gene prioritization in loci associated with complex diseases, consistent with a recent effort that showed that combining the results of several gene prioritization methods achieves better precision in gene prioritization, specifically combining different methods such as locus-based and similarity-based algorithms.[18] Future validation of the prioritized genes is needed, both with other computational methods (possibly integrating more layers of epigenetic data and expression) and also taken further

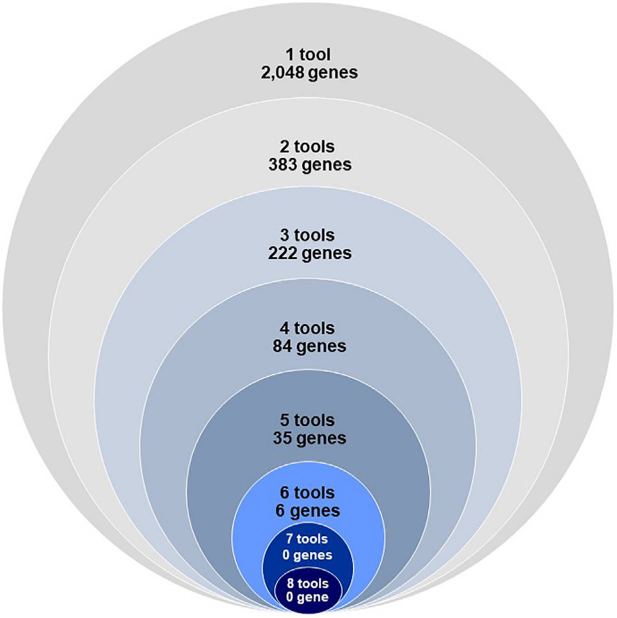

Figure 2. Number of genes prioritized by one to eight methods

to *in vitro* and *in vivo* experimental approaches. Such methods could include the deletion of candidate regulatory elements affected by the BMI-associated variants via CRISPR techniques.[81,82] Furthermore, it is important to note that the prioritization and scoring of genes depend on the availability and quality of data used by each of the prioritization methods as well as the accuracy of the methods themselves. As more reference maps of gene expression, regulatory elements, and protein levels (among other -omics) become available for more cell types and tissues and for larger sample sizes and more representative populations, the prioritization of potential causal genes in GWAS-identified loci may improve.[83]

In summary, we generated a catalog of candidate causal genes prioritized in each GWAS-identified BMI locus, which may expedite their functional characterization in experimental follow-up studies, critical to bridging the translational gap—from variant to function—which has been lacking in most GWASs.

Data and code availability

This study did not generate new datasets. The code used in this study is publicly available at GitHub (https://github.com/LoosTeam/Hemerich_BMI_gene_prioritization).

Supplemental information

Supplemental information can be found online at https://doi.org/10.1016/j.ajhg.2024.04.016.

Acknowledgments

The Novo Nordisk Foundation Center for Basic Metabolic Research is an independent research center at the University of Copenhagen, partially funded by an unrestricted donation from the Novo Nordisk Foundation (NNF18CC0034900). R.J.F.L. is supported by grants from the National Institutes of Health (R01DK124097, R01DK107786, R01DK075787, R01HL 156991, R56HG010297) and the Novo Nordisk Foundation (NNF20OC0059313). V.D.O. is supported by a grant from the Danish Diabetes and Endocrine Academy, funded by the Novo Nordisk Foundation (NNF22SA0079901).

Data were generated as part of the CommonMind Consortium supported by funding from Takeda Pharmaceuticals Company Limited, F. Hoffman-La Roche Ltd, and NIH grants (R01MH 085542, R01MH093725, P50MH066392, P50MH080405, R01MH 097276, R01MH075916, P50M096891, P50MH084053S1, R37MH 057881, R37MH057881S1, HHSN271201300031C, AG02219, AG05138, MH06692). Brain tissue was obtained from the Mount Sinai NIH Brain and Tissue Repository, the University of Pennsylvania Alzheimer's Disease Core Center, the University of Pittsburgh NeuroBioBank and Brain and Tissue Repositories, and the NIMH Human Brain Collection Core. CMC Leadership: Pamela Sklar, Joseph Buxbaum (Icahn School of Medicine at Mount Sinai), Bernie Devlin, David Lewis (University of Pittsburgh), Raquel Gur, Chang-Gyu Hahn (University of Pennsylvania), Keisuke Hirai, Hiroyoshi Toyoshiba (Takeda Pharmaceuticals Company Limited), Enrico Domenici, Laurent Essioux (F. Hoffman-La Roche Ltd), Lara Mangravite, Mette Peters (Sage Bionetworks), Thomas Lehner, and Barbara Lipska (NIMH).

We used eQTL data from The Genotype-Tissue Expression (GTEx) Project, which was supported by the Common Fund of the Office of the Director of the National Institutes of Health, and by NCI, NHGRI, NHLBI, NIDA, NIMH, and NINDS. The GTEX data used were obtained on dbGaP (phs000424.v6.p1).

Declaration of interests

The authors declare no competing interests.

Received: January 25, 2024
Accepted: April 22, 2024
Published: May 15, 2024

References

1. GBD 2015 Obesity Collaborators, Afshin, A., Forouzanfar, M.H., Reitsma, M.B., Sur, P., Estep, K., Lee, A., Marczak, L., Mokdad, A.H., Moradi-Lakeh, M., et al. (2017). Health Effects of Overweight and Obesity in 195 Countries over 25 Years. N. Engl. J. Med. *377*, 13–27.
2. NCD Risk Factor Collaboration, Abarca-Gómez, L., Abdeen, Z.A., Hamid, Z.A., Abu-Rmeileh, N.M., Acosta-Cazares, B., Acuin, C., Adams, R.J., Aekplakorn, W., Afsana, K., et al. (2017). Worldwide trends in body-mass index, underweight, overweight, and obesity from 1975 to 2016: a pooled analysis of 2416 population-based measurement studies in 128.9 million children, adolescents, and adults. Lancet *390*, 2627–2642.
3. Maes, H.H., Neale, M.C., and Eaves, L.J. (1997). Genetic and environmental factors in relative body weight and human obesity. Behav. Genet. *27*, 325–351.
4. Elks, C.E., den Hoed, M., Zhao, J.H., Sharp, S.J., Wareham, N.J., Loos, R.J.F., and Ong, K.K. (2012). Variability in the heritability of body mass index: a systematic review and meta-regression. Front. Endocrinol. *3*, 29.
5. Loos, R.J.F., and Yeo, G.S.H. (2022). The genetics of obesity: from discovery to biology. Nat. Rev. Genet. *23*, 120–133.
6. Locke, A.E., Kahali, B., Berndt, S.I., Justice, A.E., Pers, T.H., Day, F.R., Powell, C., Vedantam, S., Buchkovich, M.L., Yang, J., et al. (2015). Genetic studies of body mass index yield new insights for obesity biology. Nature *518*, 197–206.
7. Akiyama, M., Okada, Y., Kanai, M., Takahashi, A., Momozawa, Y., Ikeda, M., Iwata, N., Ikegawa, S., Hirata, M., Matsuda, K., et al. (2017). Genome-wide association study identifies 112 new loci for body mass index in the Japanese population. Nat. Genet. *49*, 1458–1467.
8. Finucane, H.K., Reshef, Y.A., Anttila, V., Slowikowski, K., Gusev, A., Byrnes, A., Gazal, S., Loh, P.R., Lareau, C., Shoresh, N., et al. (2018). Heritability enrichment of specifically expressed genes identifies disease-relevant tissues and cell types. Nat. Genet. *50*, 621–629.
9. Cano-Gamez, E., and Trynka, G. (2020). From GWAS to Function: Using Functional Genomics to Identify the Mechanisms Underlying Complex Diseases. Front. Genet. *11*, 424.
10. Wu, Y., Zeng, J., Zhang, F., Zhu, Z., Qi, T., Zheng, Z., Lloyd-Jones, L.R., Marioni, R.E., Martin, N.G., Montgomery, G.W., et al. (2018). Integrative analysis of omics summary data

reveals putative mechanisms underlying complex traits. Nat. Commun. *9*, 918.
11. Rivera, C.M., and Ren, B. (2013). Mapping human epigenomes. Cell *155*, 39–55.
12. Whalen, S., Truty, R.M., and Pollard, K.S. (2016). Enhancer-promoter interactions are encoded by complex genomic signatures on looping chromatin. Nat. Genet. *48*, 488–496.
13. ENCODE Project Consortium, Moore, J.E., Purcaro, M.J., Pratt, H.E., Epstein, C.B., Shoresh, N., Adrian, J., Kawli, T., Davis, C.A., Dobin, A., et al. (2020). Expanded encyclopaedias of DNA elements in the human and mouse genomes. Nature *583*, 699–710.
14. Andersson, R., Gebhard, C., Miguel-Escalada, I., Hoof, I., Bornholdt, J., Boyd, M., Chen, Y., Zhao, X., Schmidl, C., Suzuki, T., et al. (2014). An atlas of active enhancers across human cell types and tissues. Nature *507*, 455–461.
15. Fulco, C.P., Nasser, J., Jones, T.R., Munson, G., Bergman, D.T., Subramanian, V., Grossman, S.R., Anyoha, R., Doughty, B.R., Patwardhan, T.A., et al. (2019). Activity-by-contact model of enhancer-promoter regulation from thousands of CRISPR perturbations. Nat. Genet. *51*, 1664–1669.
16. Jung, I., Schmitt, A., Diao, Y., Lee, A.J., Liu, T., Yang, D., Tan, C., Eom, J., Chan, M., Chee, S., et al. (2019). A compendium of promoter-centered long-range chromatin interactions in the human genome. Nat. Genet. *51*, 1442–1449.
17. Pers, T.H., Karjalainen, J.M., Chan, Y., Westra, H.J., Wood, A.R., Yang, J., Lui, J.C., Vedantam, S., Gustafsson, S., Esko, T., et al. (2015). Biological interpretation of genome-wide association studies using predicted gene functions. Nat. Commun. *6*, 5890.
18. Weeks, E.M., Ulirsch, J.C., Cheng, N.Y., Trippe, B.L., Fine, R.S., Miao, J., Patwardhan, T.A., Kanai, M., Nasser, J., Fulco, C.P., et al. (2023). Leveraging polygenic enrichments of gene features to predict genes underlying complex traits and diseases. Nat. Genet. *55*, 1267–1276.
19. Yengo, L., Sidorenko, J., Kemper, K.E., Zheng, Z., Wood, A.R., Weedon, M.N., Frayling, T.M., Hirschhorn, J., Yang, J., Visscher, P.M.; and GIANT Consortium (2018). Meta-analysis of genome-wide association studies for height and body mass index in approximately 700000 individuals of European ancestry. Hum. Mol. Genet. *27*, 3641–3649.
20. Hendricks, A.E., Bochukova, E.G., Marenne, G., Keogh, J.M., Atanassova, N., Bounds, R., Wheeler, E., Mistry, V., Henning, E., Körner, A., et al. (2017). Rare Variant Analysis of Human and Rodent Obesity Genes in Individuals with Severe Childhood Obesity. Sci. Rep. *7*, 4394.
21. Marenne, G., Hendricks, A.E., Perdikari, A., Bounds, R., Payne, F., Keogh, J.M., Lelliott, C.J., Henning, E., Pathan, S., Ashford, S., et al. (2020). Exome Sequencing Identifies Genes and Gene Sets Contributing to Severe Childhood Obesity, Linking PHIP Variants to Repressed POMC Transcription. Cell Metabol. *31*, 1107–1119.e12.
22. Giambartolomei, C., Vukcevic, D., Schadt, E.E., Franke, L., Hingorani, A.D., Wallace, C., and Plagnol, V. (2014). Bayesian test for colocalisation between pairs of genetic association studies using summary statistics. PLoS Genet. *10*, e1004383.
23. Gamazon, E.R., Wheeler, H.E., Shah, K.P., Mozaffari, S.V., Aquino-Michaels, K., Carroll, R.J., Eyler, A.E., Denny, J.C., GTEx Consortium, and Nicolae, D.L., et al. (2015). A gene-based association method for mapping traits using reference transcriptome data. Nat. Genet. *47*, 1091–1098.
24. Gusev, A., Ko, A., Shi, H., Bhatia, G., Chung, W., Penninx, B.W.J.H., Jansen, R., de Geus, E.J.C., Boomsma, D.I., Wright, F.A., et al. (2016). Integrative approaches for large-scale transcriptome-wide association studies. Nat. Genet. *48*, 245–252.
25. Zhu, Z., Zhang, F., Hu, H., Bakshi, A., Robinson, M.R., Powell, J.E., Montgomery, G.W., Goddard, M.E., Wray, N.R., Visscher, P.M., and Yang, J. (2016). Integration of summary data from GWAS and eQTL studies predicts complex trait gene targets. Nat. Genet. *48*, 481–487.
26. Bycroft, C., Freeman, C., Petkova, D., Band, G., Elliott, L.T., Sharp, K., Motyer, A., Vukcevic, D., Delaneau, O., O'Connell, J., et al. (2018). The UK Biobank resource with deep phenotyping and genomic data. Nature *562*, 203–209.
27. Võsa, U., Claringbould, A., Westra, H.J., Bonder, M.J., Deelen, P., Zeng, B., Kirsten, H., Saha, A., Kreuzhuber, R., Yazar, S., et al. (2021). Large-scale cis- and trans-eQTL analyses identify thousands of genetic loci and polygenic scores that regulate blood gene expression. Nat. Genet. *53*, 1300–1310.
28. Qi, T., Wu, Y., Zeng, J., Zhang, F., Xue, A., Jiang, L., Zhu, Z., Kemper, K., Yengo, L., Zheng, Z., et al. (2018). Identifying gene targets for brain-related traits using transcriptomic and methylomic data from blood. Nat. Commun. *9*, 2282.
29. Fromer, M., Roussos, P., Sieberts, S.K., Johnson, J.S., Kavanagh, D.H., Perumal, T.M., Ruderfer, D.M., Oh, E.C., Topol, A., Shah, H.R., et al. (2016). Gene expression elucidates functional impact of polygenic risk for schizophrenia. Nat. Neurosci. *19*, 1442–1453.
30. Ng, B., White, C.C., Klein, H.U., Sieberts, S.K., McCabe, C., Patrick, E., Xu, J., Yu, L., Gaiteri, C., Bennett, D.A., et al. (2017). An xQTL map integrates the genetic architecture of the human brain's transcriptome and epigenome. Nat. Neurosci. *20*, 1418–1426.
31. Benner, C., Spencer, C.C.A., Havulinna, A.S., Salomaa, V., Ripatti, S., and Pirinen, M. (2016). FINEMAP: efficient variable selection using summary data from genome-wide association studies. Bioinformatics *32*, 1493–1501.
32. Belton, J.M., McCord, R.P., Gibcus, J.H., Naumova, N., Zhan, Y., and Dekker, J. (2012). Hi-C: a comprehensive technique to capture the conformation of genomes. Methods *58*, 268–276.
33. Watanabe, K., Taskesen, E., van Bochoven, A., and Posthuma, D. (2017). Functional mapping and annotation of genetic associations with FUMA. Nat. Commun. *8*, 1826.
34. Schmitt, A.D., Hu, M., Jung, I., Xu, Z., Qiu, Y., Tan, C.L., Li, Y., Lin, S., Lin, Y., Barr, C.L., and Ren, B. (2016). A Compendium of Chromatin Contact Maps Reveals Spatially Active Regions in the Human Genome. Cell Rep. *17*, 2042–2059.
35. Nott, A., Holtman, I.R., Coufal, N.G., Schlachetzki, J.C.M., Yu, M., Hu, R., Han, C.Z., Pena, M., Xiao, J., Wu, Y., et al. (2019). Brain cell type-specific enhancer-promoter interactome maps and disease-risk association. Science *366*, 1134–1139.
36. Itan, Y., Shang, L., Boisson, B., Ciancanelli, M.J., Markle, J.G., Martinez-Barricarte, R., Scott, E., Shah, I., Stenson, P.D., Gleeson, J., et al. (2016). The mutation significance cutoff: gene-level thresholds for variant predictions. Nat. Methods *13*, 109–110.
37. Kircher, M., Witten, D.M., Jain, P., O'Roak, B.J., Cooper, G.M., and Shendure, J. (2014). A general framework for estimating the relative pathogenicity of human genetic variants. Nat. Genet. *46*, 310–315.
38. Stenson, P.D., Mort, M., Ball, E.V., Evans, K., Hayden, M., Heywood, S., Hussain, M., Phillips, A.D., and Cooper, D.N. (2017).

The Human Gene Mutation Database: towards a comprehensive repository of inherited mutation data for medical research, genetic diagnosis and next-generation sequencing studies. Hum. Genet. *136*, 665–677.
39. de Leeuw, C.A., Mooij, J.M., Heskes, T., and Posthuma, D. (2015). MAGMA: generalized gene-set analysis of GWAS data. PLoS Comput. Biol. *11*, e1004219.
40. Gusev, A., Lawrenson, K., Lin, X., Lyra, P.C., Jr., Kar, S., Vavra, K.C., Segato, F., Fonseca, M.A.S., Lee, J.M., Pejovic, T., et al. (2019). A transcriptome-wide association study of high-grade serous epithelial ovarian cancer identifies new susceptibility genes and splice variants. Nat. Genet. *51*, 815–823.
41. Conway, J.R., Lex, A., and Gehlenborg, N. (2017). UpSetR: an R package for the visualization of intersecting sets and their properties. Bioinformatics *33*, 2938–2940.
42. Nasser, J., Bergman, D.T., Fulco, C.P., Guckelberger, P., Doughty, B.R., Patwardhan, T.A., Jones, T.R., Nguyen, T.H., Ulirsch, J.C., Lekschas, F., et al. (2021). Genome-wide enhancer maps link risk variants to disease genes. Nature *593*, 238–243.
43. Boix, C.A., James, B.T., Park, Y.P., Meuleman, W., and Kellis, M. (2021). Regulatory genomic circuitry of human disease loci by integrative epigenomics. Nature *590*, 300–307.
44. Bae-Gartz, I., Janoschek, R., Breuer, S., Schmitz, L., Hoffmann, T., Ferrari, N., Branik, L., Oberthuer, A., Kloppe, C.S., Appel, S., et al. (2019). Maternal Obesity Alters Neurotrophin-Associated MAPK Signaling in the Hypothalamus of Male Mouse Offspring. Front. Neurosci. *13*, 962.
45. Rios, M. (2013). BDNF and the central control of feeding: accidental bystander or essential player? Trends Neurosci. *36*, 83–90.
46. Huang, X., Liu, G., Guo, J., and Su, Z. (2018). The PI3K/AKT pathway in obesity and type 2 diabetes. Int. J. Biol. Sci. *14*, 1483–1496.
47. Wen, X., Zhang, B., Wu, B., Xiao, H., Li, Z., Li, R., Xu, X., and Li, T. (2022). Signaling pathways in obesity: mechanisms and therapeutic interventions. Signal Transduct. Targeted Ther. *7*, 298.
48. Huszar, D., Lynch, C.A., Fairchild-Huntress, V., Dunmore, J.H., Fang, Q., Berkemeier, L.R., Gu, W., Kesterson, R.A., Boston, B.A., Cone, R.D., et al. (1997). Targeted disruption of the melanocortin-4 receptor results in obesity in mice. Cell *88*, 131–141.
49. Yeo, G.S., Farooqi, I.S., Aminian, S., Halsall, D.J., Stanhope, R.G., and O'Rahilly, S. (1998). A frameshift mutation in MC4R associated with dominantly inherited human obesity. Nat. Genet. *20*, 111–112.
50. Vaisse, C., Clement, K., Guy-Grand, B., and Froguel, P. (1998). A frameshift mutation in human MC4R is associated with a dominant form of obesity. Nat. Genet. *20*, 113–114.
51. Farooqi, I.S., Keogh, J.M., Yeo, G.S.H., Lank, E.J., Cheetham, T., and O'Rahilly, S. (2003). Clinical Spectrum of Obesity and Mutations in the Melanocortin 4 Receptor Gene. N. Engl. J. Med. *348*, 1085–1095.
52. Gray, J., Yeo, G.S.H., Cox, J.J., Morton, J., Adlam, A.L.R., Keogh, J.M., Yanovski, J.A., El Gharbawy, A., Han, J.C., Tung, Y.C.L., et al. (2006). Hyperphagia, severe obesity, impaired cognitive function, and hyperactivity associated with functional loss of one copy of the brain-derived neurotrophic factor (BDNF) gene. Diabetes *55*, 3366–3371.
53. Kernie, S.G., Liebl, D.J., and Parada, L.F. (2000). BDNF regulates eating behavior and locomotor activity in mice. EMBO J. *19*, 1290–1300.
54. Xu, B., Goulding, E.H., Zang, K., Cepoi, D., Cone, R.D., Jones, K.R., Tecott, L.H., and Reichardt, L.F. (2003). Brain-derived neurotrophic factor regulates energy balance downstream of melanocortin-4 receptor. Nat. Neurosci. *6*, 736–742.
55. Killion, E.A., Wang, J., Yie, J., Shi, S.D.H., Bates, D., Min, X., Komorowski, R., Hager, T., Deng, L., Atangan, L., et al. (2018). Anti-obesity effects of GIPR antagonists alone and in combination with GLP-1R agonists in preclinical models. Sci. Transl. Med. *10*, eaat3392.
56. Miyawaki, K., Yamada, Y., Ban, N., Ihara, Y., Tsukiyama, K., Zhou, H., Fujimoto, S., Oku, A., Tsuda, K., Toyokuni, S., et al. (2002). Inhibition of gastric inhibitory polypeptide signaling prevents obesity. Nat. Med. *8*, 738–742.
57. Liskiewicz, A., Khalil, A., Liskiewicz, D., Novikoff, A., Grandl, G., Maity-Kumar, G., Gutgesell, R.M., Bakhti, M., Bastidas-Ponce, A., Czarnecki, O., et al. (2023). Glucose-dependent insulinotropic polypeptide regulates body weight and food intake via GABAergic neurons in mice. Nat. Metab. *5*, 2075–2085.
58. Cota, D., Proulx, K., Smith, K.A.B., Kozma, S.C., Thomas, G., Woods, S.C., and Seeley, R.J. (2006). Hypothalamic mTOR signaling regulates food intake. Science *312*, 927–930.
59. Inhoff, T., Stengel, A., Peter, L., Goebel, M., Taché, Y., Bannert, N., Wiedenmann, B., Klapp, B.F., Mönnikes, H., and Kobelt, P. (2010). Novel insight in distribution of nesfatin-1 and phospho-mTOR in the arcuate nucleus of the hypothalamus of rats. Peptides *31*, 257–262.
60. Ding, L., Zhang, L., Biswas, S., Schugar, R.C., Brown, J.M., Byzova, T., and Podrez, E. (2017). Akt3 inhibits adipogenesis and protects from diet-induced obesity via WNK1/SGK1 signaling. JCI Insight *2*, e95687.
61. Chung, W.K., Power-Kehoe, L., Chua, M., Chu, F., Aronne, L., Huma, Z., Sothern, M., Udall, J.N., Kahle, B., and Leibel, R.L. (1997). Exonic and intronic sequence variation in the human leptin receptor gene (LEPR). Diabetes *46*, 1509–1511.
62. Wu-Peng, X.S., Chua, S.C., Okada, N., Liu, S.M., Nicolson, M., and Leibel, R.L. (1997). Phenotype of the obese Koletsky (f) rat due to Tyr763Stop mutation in the extracellular domain of the leptin receptor (Lepr): evidence for deficient plasma-to-CSF transport of leptin in both the Zucker and Koletsky obese rat. Diabetes *46*, 513–518.
63. Burrell, J.A., and Stephens, J.M. (2021). KAT8, lysine acetyltransferase 8, is required for adipocyte differentiation in vitro. Biochim. Biophys. Acta, Mol. Basis Dis. *1867*, 166103.
64. Lizcano, F., Romero, C., and Vargas, D. (2011). Regulation of adipogenesis by nuclear receptor PPARgamma is modulated by the histone demethylase JMJD2C. Genet. Mol. Biol. *34*, 19–24.
65. Cho, Y.L., Min, J.K., Roh, K.M., Kim, W.K., Han, B.S., Bae, K.H., Lee, S.C., Chung, S.J., and Kang, H.J. (2015). Phosphoprotein phosphatase 1CB (PPP1CB), a novel adipogenic activator, promotes 3T3-L1 adipogenesis. Biochem. Biophys. Res. Commun. *467*, 211–217.
66. Carnevalli, L.S., Masuda, K., Frigerio, F., Le Bacquer, O., Um, S.H., Gandin, V., Topisirovic, I., Sonenberg, N., Thomas, G., and Kozma, S.C. (2010). S6K1 plays a critical role in early adipocyte differentiation. Dev. Cell *18*, 763–774.
67. Joslin, A.C., Sobreira, D.R., Hansen, G.T., Sakabe, N.J., Aneas, I., Montefiori, L.E., Farris, K.M., Gu, J., Lehman, D.M., Ober,

C., et al. (2021). A functional genomics pipeline identifies pleiotropy and cross-tissue effects within obesity-associated GWAS loci. Nat. Commun. *12*, 5253.
68. Pahl, M.C., Doege, C.A., Hodge, K.M., Littleton, S.H., Leonard, M.E., Lu, S., Rausch, R., Pippin, J.A., De Rosa, M.C., Basak, A., et al. (2021). Cis-regulatory architecture of human ESC-derived hypothalamic neuron differentiation aids in variant-to-gene mapping of relevant complex traits. Nat. Commun. *12*, 6749.
69. Stankiewicz, P., Khan, T.N., Szafranski, P., Slattery, L., Streff, H., Vetrini, F., Bernstein, J.A., Brown, C.W., Rosenfeld, J.A., Rednam, S., et al. (2017). Haploinsufficiency of the Chromatin Remodeler BPTF Causes Syndromic Developmental and Speech Delay, Postnatal Microcephaly, and Dysmorphic Features. Am. J. Hum. Genet. *101*, 503–515.
70. Perez, Y., Menascu, S., Cohen, I., Kadir, R., Basha, O., Shorer, Z., Romi, H., Meiri, G., Rabinski, T., Ofir, R., et al. (2018). RSRC1 mutation affects intellect and behaviour through aberrant splicing and transcription, downregulating IGFBP3. Brain *141*, 961–970.
71. Scala, M., Mojarrad, M., Riazuddin, S., Brigatti, K.W., Ammous, Z., Cohen, J.S., Hosny, H., Usmani, M.A., Shahzad, M., Riazuddin, S., et al. (2020). RSRC1 loss-of-function variants cause mild to moderate autosomal recessive intellectual disability. Brain *143*, e31.
72. Schneider, A., Puechberty, J., Ng, B.L., Coubes, C., Gatinois, V., Tournaire, M., Girard, M., Dumont, B., Bouret, P., Magnetto, J., et al. (2015). Identification of disrupted AUTS2 and EPHA6 genes by array painting in a patient carrying a de novo balanced translocation t(3;7) with intellectual disability and neurodevelopment disorder. Am. J. Med. Genet. *167A*, 3031–3037.
73. Sobreira, D.R., Joslin, A.C., Zhang, Q., Williamson, I., Hansen, G.T., Farris, K.M., Sakabe, N.J., Sinnott-Armstrong, N., Bozek, G., Jensen-Cody, S.O., et al. (2021). Extensive pleiotropism and allelic heterogeneity mediate metabolic effects of IRX3 and IRX5. Science *372*, 1085–1091.
74. Jastreboff, A.M., Aronne, L.J., Ahmad, N.N., Wharton, S., Connery, L., Alves, B., Kiyosue, A., Zhang, S., Liu, B., Bunck, M.C., et al. (2022). Tirzepatide Once Weekly for the Treatment of Obesity. N. Engl. J. Med. *387*, 205–216.
75. de Mesquita, Y.L.L., Pera Calvi, I., Reis Marques, I., Almeida Cruz, S., Padrao, E.M.H., Carvalho, P.E.d.P., da Silva, C.H.A., Cardoso, R., Moura, F.A., and Rafalskiy, V.V. (2023). Efficacy and safety of the dual GIP and GLP-1 receptor agonist tirzepatide for weight loss: a meta-analysis of randomized controlled trials. Int. J. Obes. *47*, 883–892.
76. Talukdar, S., Zhou, Y., Li, D., Rossulek, M., Dong, J., Somayaji, V., Weng, Y., Clark, R., Lanba, A., Owen, B.M., et al. (2016). A Long-Acting FGF21 Molecule, PF-05231023, Decreases Body Weight and Improves Lipid Profile in Non-human Primates and Type 2 Diabetic Subjects. Cell Metabol. *23*, 427–440.
77. Bhatt, D.L., Bays, H.E., Miller, M., Cain, J.E., 3rd, Wasilewska, K., Andrawis, N.S., Parli, T., Feng, S., Sterling, L., Tseng, L., et al. (2023). The FGF21 analog pegozafermin in severe hypertriglyceridemia: a randomized phase 2 trial. Nat. Med. *29*, 1782–1792.
78. Loomba, R., Sanyal, A.J., Kowdley, K.V., Bhatt, D.L., Alkhouri, N., Frias, J.P., Bedossa, P., Harrison, S.A., Lazas, D., Barish, R., et al. (2023). Randomized, Controlled Trial of the FGF21 Analogue Pegozafermin in NASH. N. Engl. J. Med. *389*, 998–1008.
79. Sasaki, T., Matsui, S., and Kitamura, T. (2016). Control of Appetite and Food Preference by NMDA Receptor and Its Co-Agonist d-Serine. Int. J. Mol. Sci. *17*, 1081.
80. Deng, S.N., Yan, Y.H., Zhu, T.L., Ma, B.K., Fan, H.R., Liu, Y.M., Li, W.G., and Li, F. (2019). Long-Term NMDAR Antagonism Correlates Weight Loss With Less Eating. Front. Psychiatr. *10*, 15.
81. Gupta, R.M., Hadaya, J., Trehan, A., Zekavat, S.M., Roselli, C., Klarin, D., Emdin, C.A., Hilvering, C.R.E., Bianchi, V., Mueller, C., et al. (2017). A Genetic Variant Associated with Five Vascular Diseases Is a Distal Regulator of Endothelin-1 Gene Expression. Cell *170*, 522–533.e15.
82. Gasperini, M., Hill, A.J., McFaline-Figueroa, J.L., Martin, B., Kim, S., Zhang, M.D., Jackson, D., Leith, A., Schreiber, J., Noble, W.S., et al. (2019). A Genome-wide Framework for Mapping Gene Regulation via Cellular Genetic Screens. Cell *176*, 1516–2390.e319.
83. Kreitmaier, P., Katsoula, G., and Zeggini, E. (2023). Insights from multi-omics integration in complex disease primary tissues. Trends Genet. *39*, 46–58.

The American Journal of Human Genetics, Volume *111*

Supplemental information

An integrative framework to prioritize genes in more than 500 loci associated with body mass index

Daiane Hemerich, Victor Svenstrup, Virginia Diez Obrero, Michael Preuss, Arden Moscati, Joel N. Hirschhorn, and Ruth J.F. Loos

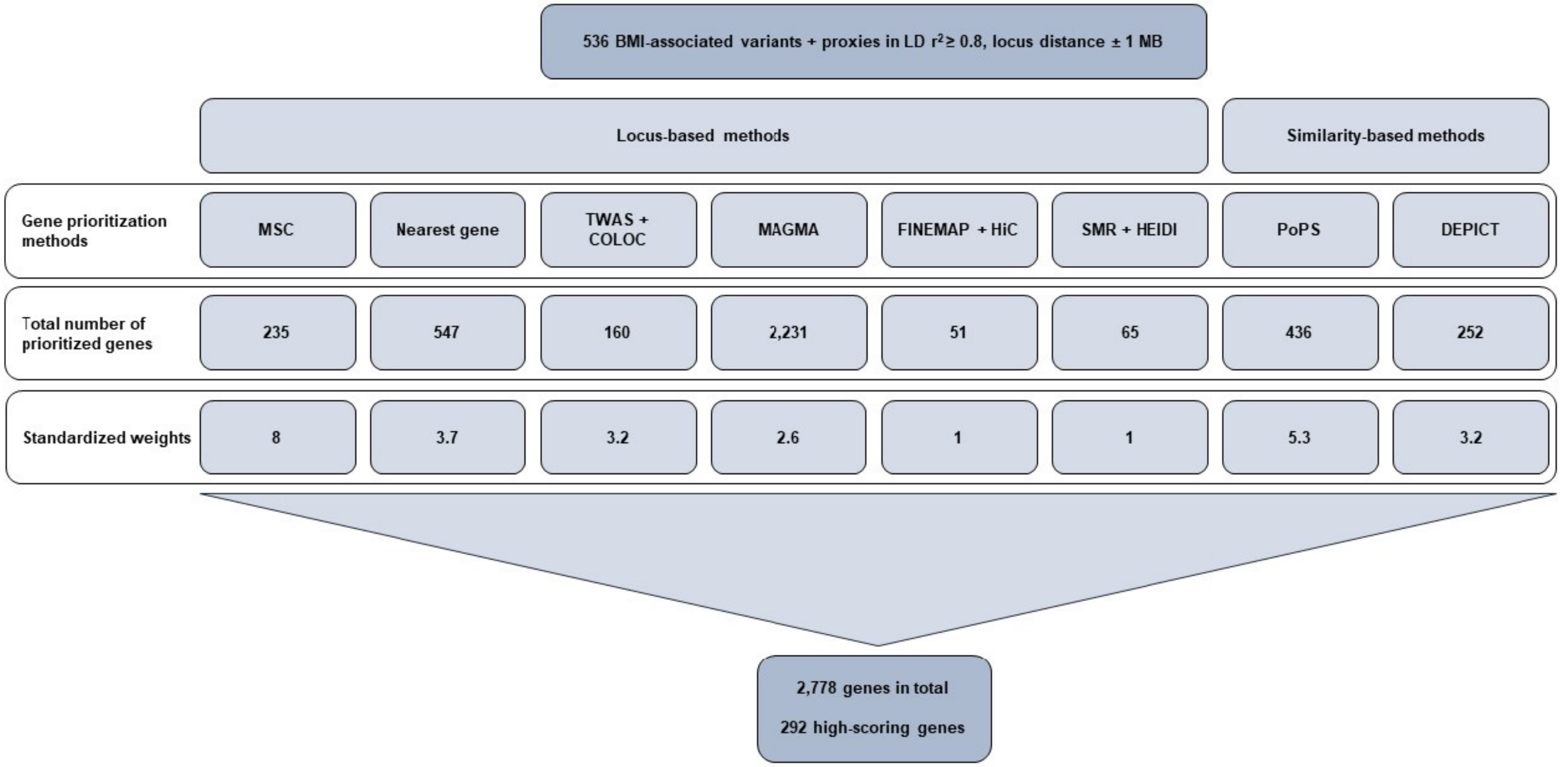

Figure S1. Flowchart of pipeline, number of genes prioritized and contribution (weights) of each method to the overall scoring of prioritized genes.
